# Smart Monitoring of Power Transformers in Substation 4.0: Multi-Sensor Integration and Machine Learning Approach

**DOI:** 10.3390/s25175469

**Published:** 2025-09-03

**Authors:** Fabio Henrique de Souza Duz, Tiago Goncalves Zacarias, Ronny Francis Ribeiro Junior, Fabio Monteiro Steiner, Frederico de Oliveira Assuncao, Erik Leandro Bonaldi, Luiz Eduardo Borges-da-Silva

**Affiliations:** 1 R&D Department, Gnarus Institute, Itajuba 37500-052, MG, Brazil; fabioduz@hotmail.com (F.H.d.S.D); tiago@institutognarus.com.br (T.G.Z.); 2 EDF Norte Fluminense, Macae 27910-970, RJ, Brazil; fabio.steiner@edfbrasil.com.br; 3 PS Soluções, Itajuba 37502-485, MG, Brazil; fred@pssolucoes.com.br (F.d.O.A.); erik@pssolucoes.com.br (E.L.B.); leborges@unifei.edu.br (L.E.B.-d.-S)

**Keywords:** fault detection, power transformers, sensor-based monitoring, online frequency response analysis, Random Forest

## Abstract

Power transformers are critical components in electrical power systems, where failures can cause significant outages and economic losses. Traditional maintenance strategies, typically based on offline inspections, are increasingly insufficient to meet the reliability requirements of modern digital substations. This work presents an integrated multi-sensor monitoring framework that combines online frequency response analysis (OnFRA^®^ 4.0), capacitive tap-based monitoring (FRACTIVE^®^ 4.0), dissolved gas analysis, and temperature measurements. All data streams are synchronized and managed within a SCADA system that supports real-time visualization and historical traceability. To enable automated fault diagnosis, a Random Forest classifier was trained using simulated datasets derived from laboratory experiments that emulate typical transformer and bushing degradation scenarios. Principal Component Analysis was employed for dimensionality reduction, improving model interpretability and computational efficiency. The proposed model achieved perfect classification metrics on the simulated data, demonstrating the feasibility of combining high-fidelity monitoring hardware with machine learning techniques for anomaly detection. Although no in-service failures have been recorded to date, the monitoring infrastructure is already tested and validated through laboratory conditions, enabling continuous data acquisition.

## 1. Introduction

Power transformers are critical components in Electrical Power Systems (EPSs), responsible for adapting voltage levels to ensure efficient energy transmission and distribution. Failures in these assets, caused by overloads, short circuits, oil leaks, insulation degradation or overvoltages, can lead to widespread outages and severe economic losses [1,2]. As modern power grids evolve toward higher levels of interconnection and renewable integration, tolerance for unplanned outages is diminishing, requiring continuous condition awareness and predictive maintenance strategies instead of traditional periodic inspections.

Traditional maintenance approaches, largely based on offline testing and visual inspections, are increasingly insufficient to meet the reliability demands of digital substations. The emerging Substation 4.0 paradigm addresses these limitations by integrating smart sensors, online diagnostic techniques, and data-driven decision-making to enable real-time health assessment and proactive asset management [3,4,5]. Implementing this vision, however, requires combining complementary monitoring techniques and advanced analytics capable of detecting subtle early-stage faults across multiple transformer subsystems.

Among the available diagnostic tools, frequency response analysis (FRA) is highly sensitive to mechanical and electrical changes in windings, cores, and internal connections. However, its conventional offline application restricts usage to scheduled maintenance windows [6,7]. Recent developments in online FRA systems overcome this limitation, enabling continuous monitoring under normal operation. Similarly, capacitive tap-based techniques provide valuable insights into bushing dielectric behavior by tracking variations in capacitance and dissipation factor. When combined with dissolved gas analysis (DGA), temperature, voltage, and current measurements, these techniques support a more comprehensive assessment of asset condition.

Despite these advances, a major challenge remains: processing and interpreting the massive amount of heterogeneous data generated by multi-sensor systems to reliably detect anomalies at an early stage. This calls for artificial intelligence (AI) methods capable of extracting meaningful patterns and identifying incipient faults that might be overlooked by conventional threshold-based diagnostics [8,9,10,11].

In this work, we address this gap by proposing an integrated methodology that combines real-time FRA and capacitive tap-based monitoring with DGA, thermal, and electrical measurements, fused into a machine learning framework for automated anomaly detection. A Random Forest (RF) classifier, enhanced with Principal Component Analysis (PCA) for dimensionality reduction, is employed to distinguish between normal and faulty conditions. The system was deployed on a Siemens transformer (model TULM 8366); however, since the installation is recent, no in-service failures have been recorded. Therefore, evaluation was carried out using controlled laboratory tests to characterize fault signatures and simulated datasets derived from these measurements. This approach provides a proof of concept for integrating high-fidelity multi-sensor hardware with AI-based diagnostics within Substation 4.0 environments.

Unlike previous transformer monitoring studies that mostly rely on conventional diagnostic data such as DGA, bushing condition, or temperature, this work introduces the novel use of online FRA measurements for transformer monitoring. While FRA has long been established as a powerful tool in laboratory or offline assessments, its application to online monitoring of power transformers is still very recent and scarcely addressed in the literature. To the authors’ knowledge, no comparable practical implementations have been reported so far. This gap reinforces the originality of the proposed approach, which combines online FRA with established indices in a machine learning framework for Substation 4.0 environments.

The remainder of this paper is organized as follows: Section 2 details the monitoring hardware and machine learning methodology. Section 3 presents and discusses the results obtained from laboratory tests and simulated anomalies. Finally, Section 4 summarizes the main findings and outlines directions for future research.

## 2. Material and Methods

This section describes the architecture and methodology adopted for implementing the online diagnostic system for power transformer and bushing condition assessment within a multi-sensor digital substation framework. The proposed approach integrates real-time frequency response analysis, capacitive tap-based monitoring, dissolved gas analysis, thermal sensing, and acquisition of electrical variables. These data are organized and made available by a SCADA system, enabling the application of a machine learning algorithm for automated health assessment.

### 2.1. Equipment and General Characteristics

The monitored asset is a large three-phase power transformer manufactured by Siemens, model TULM 8366, designed for outdoor installation. It was manufactured in 2002 and, according to its nameplate, the general and specific characteristics are summarized in Table 1. The transformer includes integrated current transformers in the bushings, models 10B400 and JC500, and uses capacitive bushings installed at terminals T11 to T42.

The monitoring infrastructure integrates several modules installed on the transformer body, bushing taps, and associated control panels. All devices communicate through a structured industrial network using Modbus/TCP and OPC-UA protocols. The monitoring architecture employs a dedicated merging unit that consolidates the OnFRA^®^ 4.0 and FRACTIVE^®^ 4.0 modules, integrating them with the Siemens SITRAM Multisense system for dissolved gas and temperature monitoring. Additionally, this unit receives input from the programmable logic controller (PLC), which provides voltage, current, and power measurements from the transformer.

Acting as a central hub, the merging unit manages and synchronizes all monitoring signals. Multiprotocol converters, Ethernet switches, and optical fiber transceivers are used to ensure galvanic isolation and high immunity to electromagnetic interference. This configuration allows for seamless data acquisition and secure communication among sensors, enabling reliable transmission of frequency response, bushing condition, thermal behavior, and electrical parameters to the SCADA platform for both real-time visualization and historical analysis.

### 2.2. Online Frequency Response Module—OnFRA ^®^ 4.0

**Figure 1:**
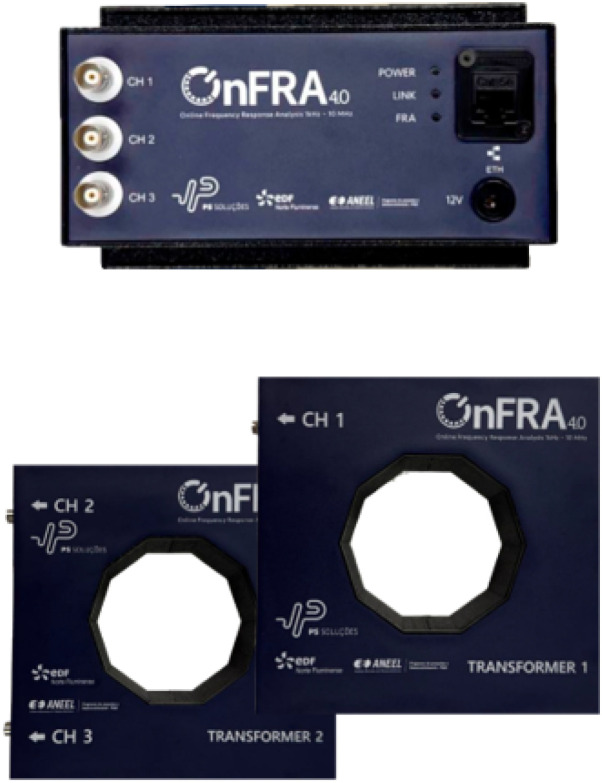
OnFRA^®^ 4.0 device.

OnFRA^®^ 4.0 is a device developed for real-time frequency response analysis (FRA) of power transformers during live operation, eliminating the need for service interruption, as shown in Figure 1. The system injects a high-frequency sinusoidal signal into the winding under test and measures the corresponding electrical response through precision voltage and current sensors. The transfer impedance:

(1) $$Z\left(f\right)=\frac{V(f)}{I(f)}$$

is computed and compared against reference curves to identify deviations associated with insulation degradation or mechanical faults. In contrast to conventional offline FRA techniques, OnFRA^®^ 4.0 supports continuous monitoring under load conditions by employing galvanically isolated injection and acquisition modules, which ensure signal integrity and safe operation on energized equipment [12,13,14].

Beyond supporting both online and offline insulation diagnostics, the platform provides a wideband frequency response covering 100 Hz to 10 MHz and simultaneously measures impedance, capacitance, resistance, and the dissipation factor (tanδ). It interfaces seamlessly with the Preditor^®^ platform via OPC UA (Client/Server) and enables detection of localized anomalies in transformer cores, windings, and inter-turn insulation. The system is also compatible with oil-immersed and dry-type transformers, as well as motors, generators, and power cables.

The OnFRA^®^ 4.0 system detects degradation scenarios that alter the frequency-domain electrical behavior of transformer windings. These include inter-turn short circuits, coil loosening or displacement, axial or radial deformation, dielectric moisture ingress and thermal aging, core-clamping deterioration, and open-circuit conditions in specific winding sections. Such failure mechanisms manifest as distinct signatures in the impedance magnitude and phase curves across specific frequency bands.

For field installation, the system employs coupling elements comprising current and voltage injection and measurement transformers connected directly to transformer terminals. Its architecture integrates a high-frequency signal generator, differential voltage sensors, Rogowski-type current coils, wideband signal conditioning hardware, and a digital control module interfaced with the Preditor^®^ environment. This configuration enables automated report generation and real-time visualization of impedance, phase, and diagnostic indices.

All acquired spectra are stored in the Preditor^®^ database, where they can be visualized through time-stamped trend curves of Z(f), phase, resistance, capacitance, and the dissipation factor (tanδ). The platform also provides deviation metrics such as the Absolute Sum of Logarithmic Errors (ASLE), supports baseline comparisons across historical datasets or between phases, and issues automated alerts when statistical deviations or signature shifts are detected. These features ensure full historical traceability and facilitate predictive maintenance strategies.

To aid in the diagnostic process, Table 2 summarizes common winding failure modes detectable by OnFRA^®^ 4.0, including their typical frequency ranges and characteristic patterns observed in impedance magnitude (|Z(f)|) and phase angle (∠Z(f)) responses, following IEEE C57.149™–2012 [15].

### 2.3. Capacitive Tap-Based Monitoring—FRACTIVE^®^ 4.0

**Figure 2:**
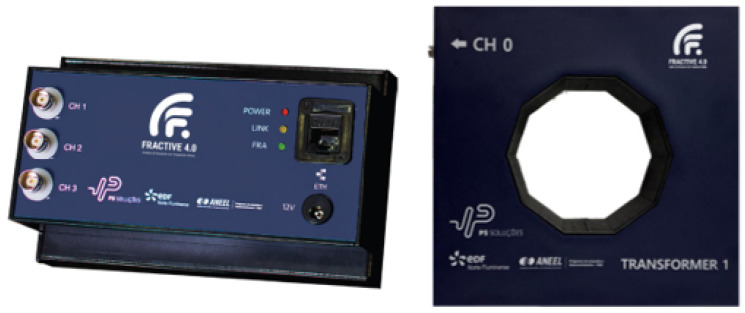
Fractive^®^ 4.0 device.

FRACTIVE^®^ 4.0 continuously monitors high-voltage capacitive bushings using Sweep Frequency Response Analysis (SFRA), as shown in Figure 2. It is the first system to apply FRA techniques in load conditions without requiring equipment shutdown, allowing real-time assessment of bushing health. Moving beyond traditional leakage current methods, FRACTIVE^®^ 4.0 detects subtle dielectric and mechanical anomalies indicative of early-stage insulation degradation.

The system operates by injecting controlled high-frequency voltage signals into the bushing test tap (commonly terminal H0) and measuring the resulting current and voltage responses. The complex impedance spectrum is then computed as:

(2) $$Z\left(f\right)\frac{V_{\mathrm{FRA}}\left(f\right)}{I_{\mathrm{FRA}}\left(f\right)}$$

which is analyzed over time and compared with baseline measurements to detect deviations associated with progressive structural deterioration [16].

FRACTIVE^®^ 4.0 provides a set of key features that enable online and real-time frequency response analysis of capacitive bushings during transformer operation, eliminating the need for disconnection. The system covers an extended frequency range from 1 kHz to 10 MHz, offering high-resolution analysis of both time-domain and frequency-domain responses [17]. It integrates seamlessly with the Preditor^®^ platform via OPC UA, supporting visualization, event logging, and automated reporting. Diagnostic indicators include calculated dielectric parameters such as capacitance ($C_{p}$), resistance ($R_{p}$), and dissipation factor (tanδ), as well as statistical deviation metrics like the Absolute Sum of Logarithmic Errors (ASLE) [12].

The device is capable of detecting and classifying a wide range of incipient and advanced fault conditions in bushings. These include delamination of capacitive layers, gas pockets or voids between dielectric layers, partial insulation breakdown, capacitive unbalance across phases, loose terminal connections, and carbon tracking or surface contamination. Such faults manifest as localized variations in the impedance spectrum, often restricted to specific frequency bands. To facilitate diagnosis, FRACTIVE^®^ 4.0 segments the frequency response into low-, mid-, and high-frequency regions, correlating observed anomalies with underlying physical degradation mechanisms.

In terms of system integration, each FRACTIVE^®^ 4.0 unit can simultaneously monitor multiple bushings, typically H1, H2, H3, and H0, using a High-Frequency Current Transformer (HFCT) to capture return currents in the ground path with minimal distortion. The system architecture comprises signal injection and measurement hardware, a current measurement block with HFCT and linearization circuitry, a control unit responsible for signal generation and synchronization, and an analysis module embedded within the Preditor^®^ supervisory software.

All acquired data are time-stamped, stored in SQL Server databases, and visualized via an Elipse E3-based supervisory interface. The platform supports tracking of dielectric parameter trends over time, tri-phase impedance asymmetry, and real-time alerts based on threshold exceedance or statistical divergence criteria, such as ASLE values exceeding 10%.

### 2.4. Dissolved Gas Analysis and Thermal Monitoring

**Figure 3:**
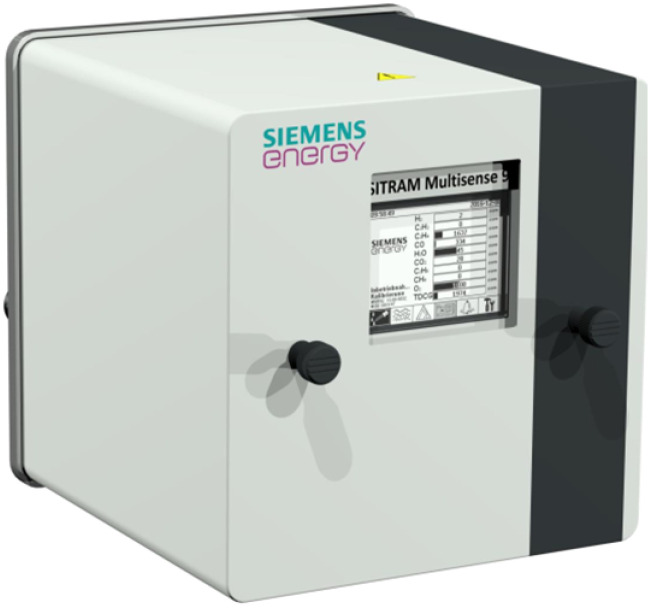
Siemens SITRAM Multisense DGA and temperature monitoring system.

The Siemens SITRAM Multisense system (Figure 3) provides continuous monitoring of key dissolved gases in transformer oil, including $H_{2}$, CO, $C_{2}H_{2}$, and ${\mathrm{CH}}_{4}$, as well as oil and ambient temperatures. This multi-parameter approach enables early detection of incipient faults such as arcing, overheating, and insulation breakdown. Gas concentrations are measured using a combination of infrared and electrochemical sensors equipped with automatic calibration routines, ensuring measurement stability over extended operation. Data are transmitted via Modbus/TCP to the SCADA system at one-minute intervals, supporting near real-time supervision. Beyond standalone diagnostics, this information is particularly valuable when correlated with frequency response anomalies or bushing parameter drifts, forming part of the input dataset used in the supervised learning model for fault detection and predictive maintenance [18,19].

### 2.5. Supervisory System and Data Management

A dedicated SCADA system, developed using Elipse E3, aggregates, visualizes, and manages all monitoring variables within a unified graphical interface. Each incoming sensor data stream is timestamped, stored in a SQL Server database, and subsequently made available for real-time dashboards, historical trend analysis, and alarm management. The data architecture is organized into three layers: raw tables, which contain direct values from each sensor, such as bushing capacitance, frequency response analysis (FRA) curves, and dissolved gas levels; processed tables, which store calculated indices, normalized trends, and classification results generated by the diagnostic algorithms; and log tables, which record timestamped alarms, operating status, and communication health to support cybersecurity auditing. This structured SQL design enables full traceability and data lineage analysis, providing deterministic decision-making capabilities and facilitating seamless model retraining when new operational conditions arise.

All monitoring modules are synchronized through network protocol timestamping. The SCADA system integrates data fusion routines that guarantee proper temporal alignment across devices with heterogeneous sampling rates [20]. For example, FRA and capacitive measurements are acquired at intervals of 5 to 30 min, dissolved gas analysis (DGA) and temperature readings are updated every 1 to 5 min, and electrical quantities are sampled every 30 s. This multi-resolution approach ensures consistency in the monitoring framework and supports reliable condition assessment of the asset.

### 2.6. Machine Learning-Based Diagnostic Algorithm

The diagnostic module is implemented in Python and deployed as a service that directly interfaces with the SQL Server database through pyodbc and SQL queries. Its core algorithm relies on the Random Forest (RF) technique, trained using historical labeled datasets encompassing both degradation scenarios and normal operating conditions. The model leverages a set of features that capture the most relevant patterns for transformer health assessment, including the rate of capacitance variation, the slope of the tangent delta, shifts in frequency band resonance, gas concentration trends, temperature differentials, and asymmetry indices. These parameters collectively enhance the model’s ability to identify incipient faults and improve the reliability of the overall monitoring framework.

#### 2.6.1. Random Forest

Random Forest is a supervised machine learning algorithm based on ensemble learning, introduced by Breiman [21]. It combines multiple decision trees to improve predictive performance and control overfitting. RF is widely used for classification and regression tasks, including fault diagnosis in electrical power systems.

Each tree in a Random Forest is usually trained using a bootstrap sample from the original dataset, and a random subset of features is selected at each split. This randomness introduces diversity among the trees, making the ensemble more robust.

Let $D={\left\{\left(x_{i},y_{i}\right)\right\}}_{i=1}^{n}$ be a training dataset where $x_{i}\in R^{d}$ represents the feature vector and $y_{i}$ the class label (e.g., fault type). The prediction of the Random Forest is given by:

(3) $$\hat{y}=\mathrm{mode}{\left\{h_{t}\left(x\right)\right\}}_{t=1}^{T},$$

where $h_{t}\left(x\right)$ is the prediction of the *t*-th decision tree, and *T* is the total number of trees in the forest. For regression tasks, the final output is:

(4) $$\hat{y}=\frac{1}{T}\sum_{t=1}^{T}h_{t}\left(x\right).$$

Random Forest is particularly suitable for fault detection due to several key characteristics. Its robustness to overfitting is one of its main strengths, as the ensemble averaging process reduces variance compared to individual decision trees. Furthermore, it excels at capturing nonlinear patterns, allowing it to model complex relationships between operational variables and different failure modes. Another important advantage is its ability to estimate feature importance, which helps identify the variables most strongly associated with faults. Finally, Random Forest tends to achieve high accuracy when applied to labeled datasets, especially in scenarios where fault types are well-defined and properly annotated.

**Figure 4:**
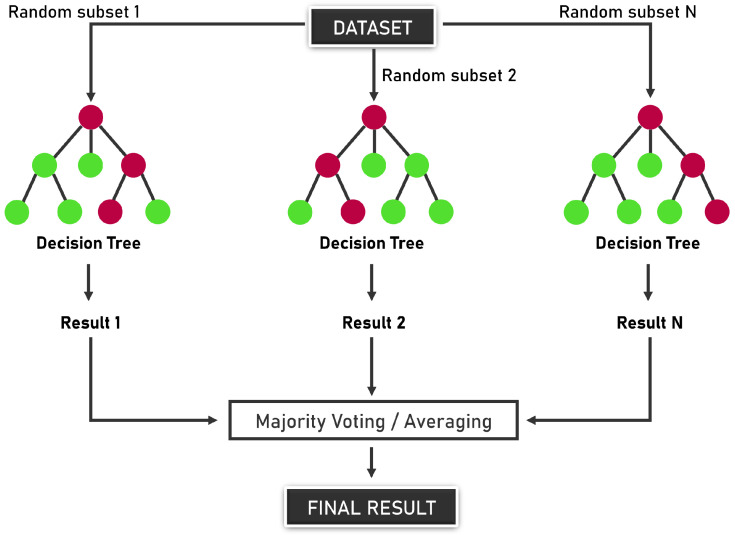
Random Forest model. The red nodes represent decision splits, while the green nodes represent terminal (leaf) nodes corresponding to class predictions.

The overall structure of a Random Forest model, with multiple decision trees combined to form a robust classifier, is illustrated in Figure 4.

#### 2.6.2. Principal Component Analysis

Principal Component Analysis is a widely used statistical technique for dimensionality reduction, originally introduced by Pearson [22] and later formalized by Hotelling [23]. The primary objective of PCA is to transform a set of possibly correlated variables into a new set of uncorrelated variables, known as principal components.

This method seeks an orthogonal basis that maximizes the variance explained by the data, preserving as much information as possible while reducing the number of dimensions. Dimensionality reduction is particularly relevant in electrical systems and industrial applications, where high-dimensional datasets can hinder modeling efforts, increase the risk of overfitting, and raise computational costs [24].

Given a dataset $X\in R^{n\times d}$, with *n* observations and *d* variables, the PCA procedure can be described as follows [24]:

1. **Data centralization:** Subtract the mean of each variable from the dataset, obtaining the centered matrix $X_{\mathrm{centered}}$.
2. **Covariance matrix computation:** Calculate the sample covariance matrix as
  (5) $$\Sigma =\frac{1}{n-1}X_{\mathrm{centered}}^{⊤}X_{\mathrm{centered}}.$$
3. **Spectral decomposition:** Perform eigen decomposition of the covariance matrix Σ to obtain the eigenvalues $\lambda_{i}$ and their corresponding eigenvectors $v_{i}$.
4. **Selection of principal components:** Choose the k<d eigenvectors associated with the largest eigenvalues, such that the cumulative explained variance exceeds a desired threshold (e.g., 80%).
5. **Projection onto principal component space:** Project the centered data onto the new basis formed by the selected eigenvectors:
  (6) $$Z=X_{\mathrm{centered}}V_{k},$$
  where $V_{k}$ is the matrix whose columns are the *k* selected eigenvectors.

The proportion of variance explained by each principal component *i* is given by

(7) $${\mathrm{Var}}_{i}=\frac{\lambda_{i}}{\sum_{j=1}^{d}\lambda_{j}}.$$

which represents the proportion of total variance captured by each principal component.

#### 2.6.3. Procedures of Proposed Method

Random Forest (RF) was employed as a supervised classification model. The overall procedure involved three main steps: data preprocessing, dimensionality reduction, and hyperparameter optimization.

1. Data Preprocessing

The dataset corresponds to ten years of simulated operational data from a power transformer, including both normal and anomalous operating conditions. Each record is associated with a timestamp and an anomaly label. The monitored variables cover a wide range of electrical, thermal, chemical, and frequency response features, which are summarized as follows:

- Electrical measurements: Primary and secondary voltages and currents ($V_{\mathrm{prim},\mathrm{rms}}$, $V_{\sec,\mathrm{rms}}$, $I_{\mathrm{prim},\mathrm{rms}}$, $I_{\sec,\mathrm{rms}}$).
- Thermal conditions: Oil temperature and ambient temperature ($T_{\mathrm{oil}}$, $T_{\mathrm{env}}$).
- Dissolved gases in oil: Concentrations of hydrogen (H_2_), methane (CH_4_), acetylene (C_2_H_2_), and carbon monoxide (CO), expressed in ppm.
- Frequency response analysis (FRA): Magnitude and phase of impedance, capacitance, and tangent delta ($C_{p}$, |Z|, ∠Z, TgD) evaluated across four frequency bands (100 Hz–1 kHz, 1 kHz–10 kHz, 10 kHz–100 kHz, 100 kHz–1 MHz).
- Active FRA (FRACTIVE): Same set of FRA features measured separately on phases A, B, and C.

Altogether, the dataset contains more than 60 descriptive features plus the timestamp and anomaly columns. The feature matrix X was constructed by removing timestamp and anomaly, and Z-score normalization was applied to ensure zero mean and unit variance for each feature:

(8) $$z_{i}=\frac{x_{i}-\mu }{\sigma }$$

where $x_{i}$ is a feature value, μ the mean, and σ the standard deviation.

2. Dimensionality Reduction

To mitigate computational cost and redundancy among highly correlated variables, Principal Component Analysis (PCA) was applied. The number of retained components *k* was chosen such that at least 20% of the total variance was preserved:

(9) $$\sum_{i=1}^{k}\lambda_{i}\geq 0.20$$

where $\lambda_{i}$ denotes the *i*-th eigenvalue of the covariance matrix. This threshold was selected as a compromise between model accuracy and computational efficiency. Given the large number of monitored variables, processing the full feature set would significantly increase training cost. Therefore, an arbitrary retention of 20% variance was adopted in this study to accelerate learning while maintaining representative information. It should be noted that this choice is case-specific; in other applications, a higher retention ratio may be required. The resulting reduced feature matrix $X_{\mathrm{PCA}}\in R^{n\times k}$ was then used for classification.

3. Hyperparameter Tuning

A Grid Search with 5-fold Stratified Cross-Validation was conducted to identify the best hyperparameters, following standard machine learning practices [25,26]. The following grid was explored: number of trees ($n_{\mathrm{estimators}}\in \left\{50,100,200\right\}$), splitting criterion (gini or entropy), minimum samples per split ({2,5,10}), minimum samples per leaf ({1,2,4}), maximum features ($\sqrt{d}$, $\log_{2}$, or *d*), and bootstrap (True or False).

Due to the class imbalance, the F1-score was used as the evaluation metric during cross-validation. The best configuration from the grid search was used to train the final Random Forest model on the full dataset. For the Random Forest model, the optimal hyperparameter configuration identified was $n_{\mathrm{estimators}}=200$, criterion=gini, min_samples_split=2, min_samples_leaf=1, max_features=d, and bootstrap=True.

## 3. Results and Discussion

In this section, we present and discuss the results obtained from both the measurement system and the proposed diagnostic methods. In addition to the laboratory evaluations, the results include the implementation of the monitoring architecture in the field, covering the installation process and the integration with the SCADA supervisory platform.

As the monitored installations are relatively new, no significant fault events have been recorded to date. Consequently, part of the performance assessment is carried out under controlled laboratory conditions, where representative fault scenarios such as oil moisture, poor bushing contact and insulation degradation are artificially reproduced to analyze sensor responses under different stress conditions. The field results demonstrate the effectiveness of the deployed system in providing continuous data acquisition and real-time visualization capabilities, validating its readiness for long-term operational monitoring.

For the machine learning model, a simulated dataset is employed. This dataset is generated based on the normal operating values measured from the equipment, combined with proportional variations observed during the laboratory fault simulations. This approach allows assessing the algorithm’s ability to detect anomalies even in the absence of field failures, providing an initial validation framework that can be progressively refined as real operational data become available.

### 3.1. Field Implementation and Supervisory System Results

#### 3.1.1. Field Installation Overview

The proposed monitoring architecture was deployed in a high-voltage power transformer to validate its performance under real operational conditions and to verify the feasibility of continuous data acquisition and integration with the supervisory control system. The installation aimed to replicate the architecture previously tested in laboratory conditions, now accounting for the environmental and electromagnetic constraints present in an operational substation.

The field setup comprises three main elements:

- Merging and Diagnostic Modules: A dedicated panel was assembled to house the OnFRA^®^ 4.0 and FRACTIVE^®^ 4.0 modules. These units perform frequency response analysis and bushing condition diagnostics, respectively, and are interconnected through multiprotocol communication interfaces. The physical layout was optimized to minimize signal interference and ensure galvanic isolation between measurement channels.
- Gas and Temperature Sensing: The Siemens SITRAM Multisense sensor was installed directly on the transformer oil circuit. This sensor enables continuous measurement of key dissolved gases and oil temperature, which are crucial indicators for detecting early-stage insulation degradation, arcing phenomena, or overheating.
- Electrical Parameter Acquisition: A programmable logic controller (PLC) panel was integrated to capture voltage, current, and power measurements. These electrical parameters provide complementary information that allows correlation between mechanical and thermal stresses detected by the diagnostic modules and actual load variations in the transformer.

**Figure 5:**
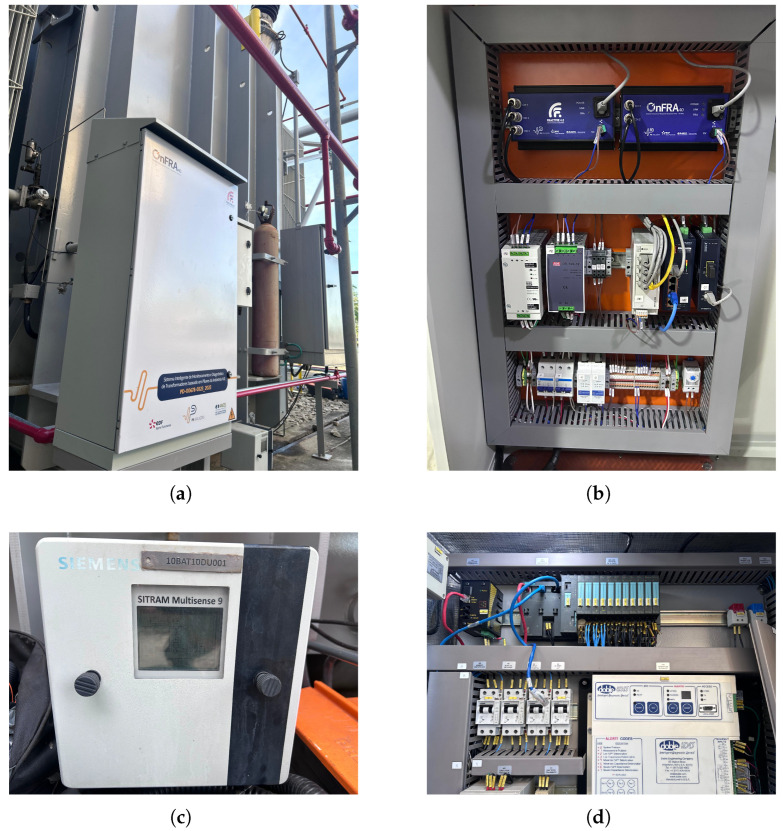
Field Implementation of the monitoring system components, including diagnostic modules, gas sensors, and electrical parameter acquisition panel. (**a**) Overview of the installed monitoring panel within the transformer bay. (**b**) Details of the OnFRA^®^ 4.0 and FRACTIVE^®^ 4.0 modules used for frequency response and bushing condition diagnostics. (**c**) Siemens SITRAM Multisense sensor installed for dissolved gas and temperature monitoring. (**d**) A programmable logic controller (PLC) panel is responsible for voltage, current, and power acquisition.

The installation process also involved the design and routing of communication links between the panels and the SCADA system. Ethernet switches and optical fiber transceivers were used to ensure robust data transmission with immunity to electromagnetic interference, a common challenge in high-voltage environments. Figure 5 shows the assembled diagnostic panel and the gas sensor installed, as well as the electrical measurement cabinet based on PLC.

#### 3.1.2. SCADA Supervisory Interface

The SCADA platform was configured to integrate and visualize all data streams from the monitoring system, providing a unified interface for real-time transformer health assessment. The supervisory interface is organized into multiple layers:

- Individual Parameter Visualization: Dedicated screens display detailed data for each subsystem, including gas concentration trends, temperature evolution, voltage and current waveforms, and results from frequency response and bushing condition analyses.
- Aggregated Transformer Status: A central dashboard consolidates these indicators, allowing operators to identify abnormal conditions at a glance. Color-coded alarms and thresholds facilitate rapid decision-making in case of incipient faults.
- Historical Data and Trend Analysis: The system stores measurement data for long-term analysis, enabling correlation of operating conditions with potential degradation patterns. This historical layer is fundamental for predictive maintenance strategies, as it supports anomaly detection algorithms and life-cycle modeling.

**Figure 6:**
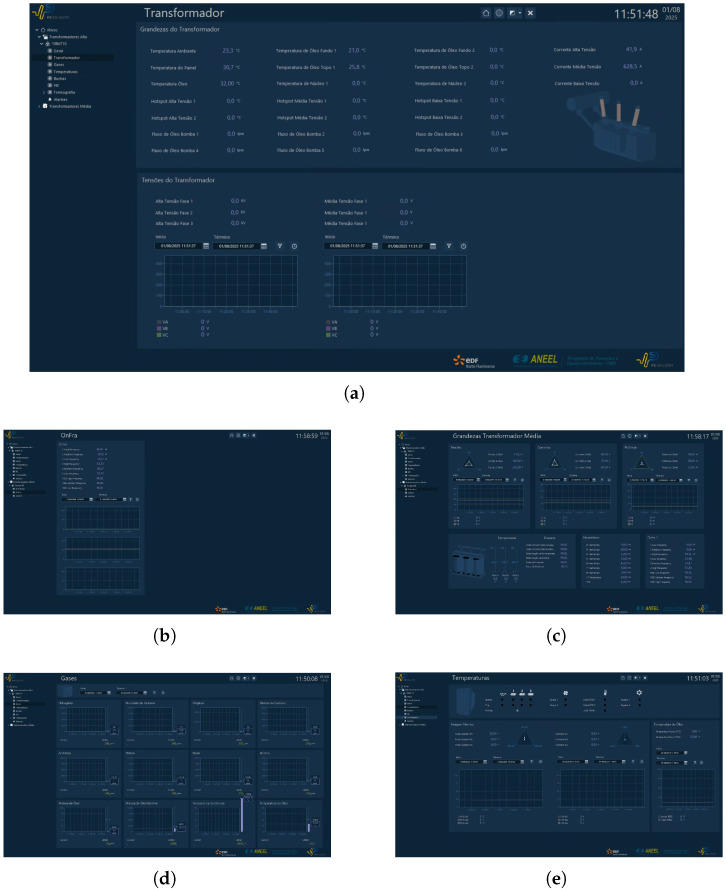
Screenshots of the SCADA supervisory interface showing real-time and historical monitoring data for the power transformer, including gas concentration, temperature, electrical parameters, and frequency response analysis. (**a**) General overview of the SCADA interface displaying the monitored transformer and its main diagnostic parameters. (**b**) Visualization of frequency response analysis results from the OnFRA^®^ module. (**c**) Electrical measurements dashboard with voltage, current, and power measurements. (**d**) Real-time dissolved gas concentration monitoring screen. (**e**) Oil temperature monitoring interface, indicating thermal behavior during operational conditions.

Figure 6 shows representative screenshots of the supervisory interface. One image highlights the visualization of dissolved gas and temperature measurements, while another provides an overview of electrical parameters and the frequency response analysis results. Together, these visualizations demonstrate the ability of the platform to centralize heterogeneous diagnostic information and support both real-time and retrospective evaluations.

The combined field and supervisory results confirm the practicality of the monitoring architecture. Despite the absence of major fault events in the monitored transformer, the system proved capable of continuously acquiring and synchronizing measurements from different diagnostic domains. Furthermore, the SCADA integration establishes a scalable framework, allowing additional sensors or analytical modules to be incorporated with minimal hardware or software modifications.

#### 3.1.3. Laboratory Validation of OnFRA^®^ 4.0

**Figure 7:**
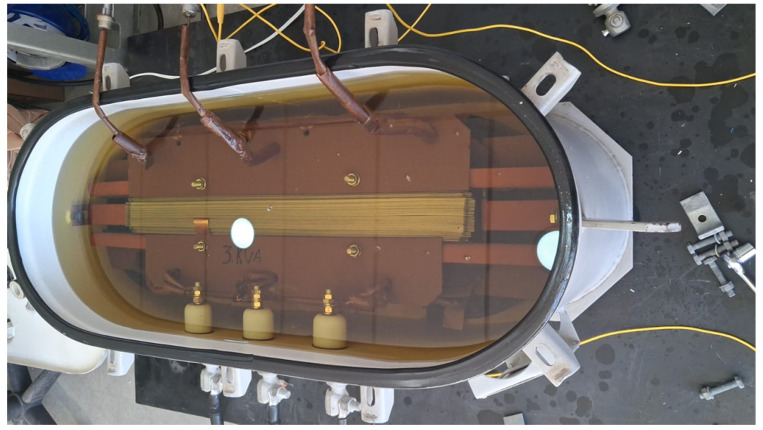
Laboratory setup for fault simulation in a 3 kVA, 13.8 kV oil-immersed transformer monitored by OnFRA^®^ 4.0. The test configuration includes high-frequency injection and acquisition coils connected to the high-voltage bushings.

To demonstrate the diagnostic capabilities of the OnFRA^®^ 4.0 system, a laboratory test was conducted on an oil-immersed transformer rated at 3 kVA and 13.8 kV, as shown in Figure 7. A controlled inter-turn short circuit was introduced in one of the primary windings to emulate insulation failure conditions. The OnFRA^®^ 4.0 system was connected to the high-voltage bushings using high-frequency injection and acquisition coils. Baseline frequency response measurements were first collected under normal operating conditions, followed by additional measurements after fault insertion.

The comparison between baseline and fault conditions revealed measurable deviations primarily within the mid-frequency range (10 kHz–1 MHz). In this range, localized inter-turn short circuits caused a progressive attenuation of the impedance magnitude, with a noticeable flattening of resonance peaks and displacement of anti-resonance points. These effects are physically associated with the reduction in the effective inductive coupling between turns and the emergence of parasitic conductive paths.

Additionally, in the low-frequency region (1 kHz–10 kHz), slight shifts in resonance peaks were observed, indicating early signs of winding mechanical displacement. At higher frequencies (>1 MHz), small ripple components appeared in the impedance magnitude, suggesting localized dielectric disturbances. These frequency-domain signatures were accompanied by phase shifts consistent with the IEEE C57.149™–2012 diagnostic criteria, reinforcing the correlation between the observed spectral changes and incipient inter-turn faults.

Quantitatively, during the inter-turn short circuit simulation on the 3 kVA transformer, the impedance magnitude in the 100 kHz–300 kHz band dropped by approximately 11–15%, while the phase curve exhibited a shift of about 8°. The resonance in the impedance chart shifted from 4.5 kHz to 5 kHz, a deviation of about 11–12%.

**Figure 8:**
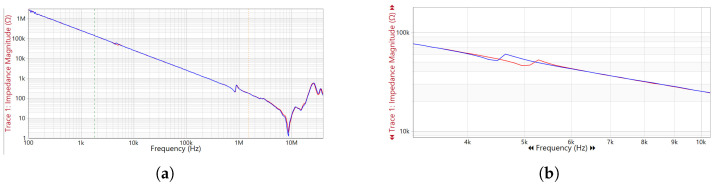
Frequency response results obtained with OnFRA^®^ 4.0 during laboratory fault simulation. The red curve represents the baseline condition (normal operation), while the blue curve corresponds to the response under fault conditions. Subfigure (**a**) presents the global impedance response, and subfigure (**b**) details the mid-frequency region where the most significant changes occur due to the controlled inter-turn short circuit. (**a**) Overall frequency response spectrum comparing normal and fault conditions. (**b**) Zoomed view highlighting deviations in resonance and anti-resonance behavior after fault insertion.

To quantify these deviations, the system automatically computed the Absolute Sum of Logarithmic Errors (ASLE) between the baseline and the fault spectrum, exceeding the threshold of 10 and triggering an anomaly alarm. Figure 8 illustrates the frequency response comparison between normal and fault conditions. Figure 8a presents the overall spectrum, while Figure 8b highlights the mid-frequency range, where the most significant deviations are observed. These results confirm that OnFRA^®^ 4.0 is capable of detecting subtle winding insulation degradation through measurable changes in impedance amplitude, resonance and anti-resonance positioning, and mid-frequency response damping, providing a non-invasive and continuous diagnostic capability to enhance transformer reliability and reduce unplanned outages.

### 3.2. Fault Simulation in Capacitive Bushing Monitored by FRACTIVE^®^ 4.0

**Figure 9:**
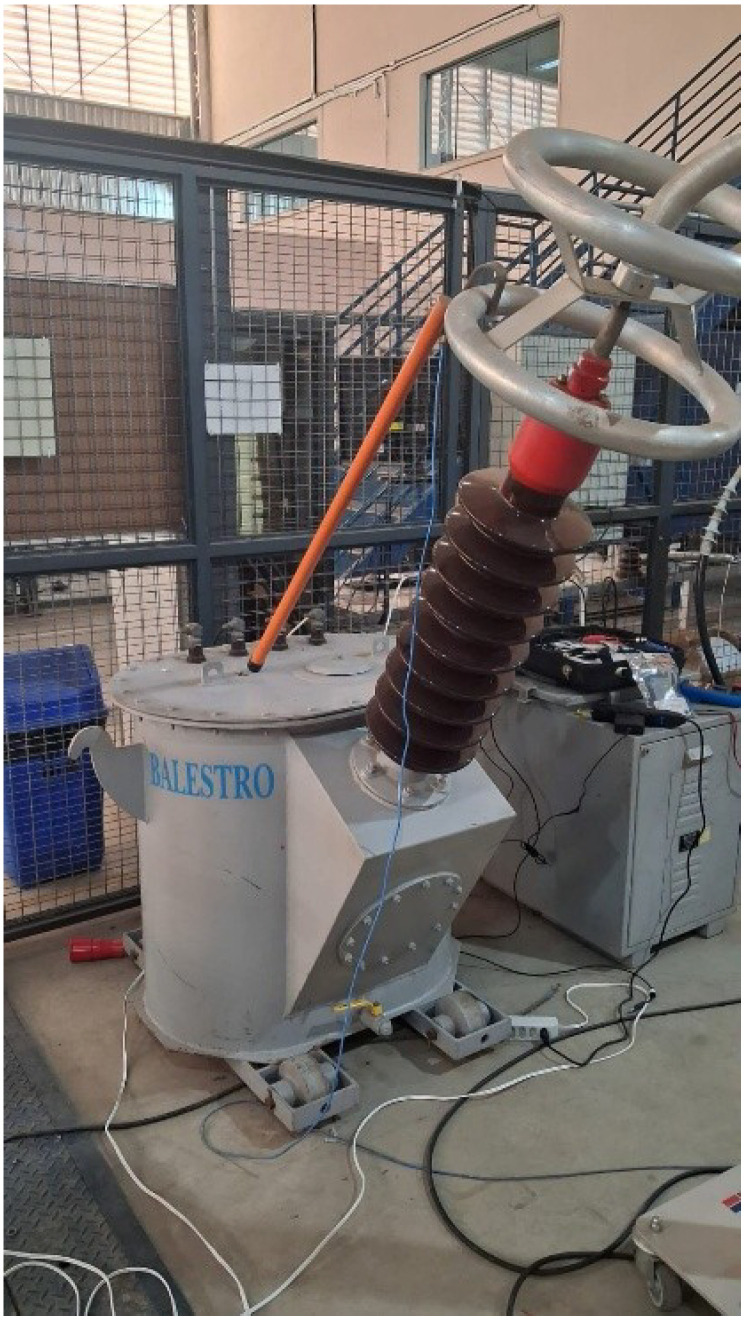
Capacitive bushing type GOB 380 (ABB) used for fault simulation.

The FRACTIVE^®^ 4.0 system enables real-time monitoring of bushing insulation by acquiring the frequency response from the test tap. In this study, a capacitive bushing type GOB 380 from ABB (100 kV, 800 A), with $C_{1}=135$ pF and tanδ=0.378% at 10 kV (Figure 9), was used for degradation simulation. The FRACTIVE^®^ 4.0 was connected via a long shielded cable to the test tap, enabling non-invasive impedance and admittance measurements over a broad frequency spectrum.

**Figure 10:**
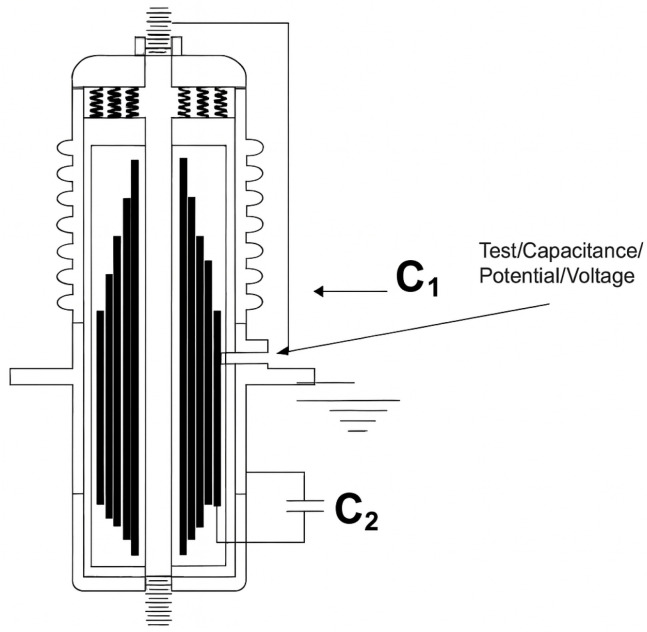
Simplified capacitive divider equivalent of the GOB 380 bushing: $C_{1}$ (core-to-layer capacitance) and $C_{2}$ (layer-to-ground capacitance).

To emulate degradation in the capacitance path $C_{2}$, commonly associated with moisture ingress or surface contamination, series capacitors of 4.7 nF, 8.0 nF, and 10.0 nF were connected between the test tap and the ground. These additions simulate leakage paths or internal insulation faults, modifying the capacitive divider formed by $C_{1}$ and $C_{2}$ in the bushing structure. The simplified equivalent circuit is illustrated in Figure 10, where $C_{1}$ represents the capacitance between the central conductor and internal conductive layers, and $C_{2}$ corresponds to the capacitance between the last conductive layer and ground.

**Figure 11:**
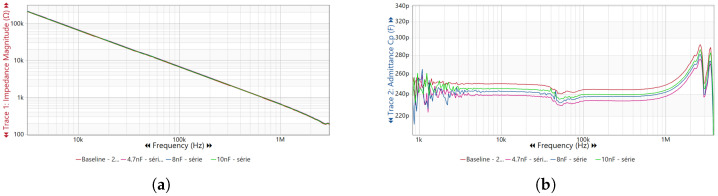
Frequency response of the capacitive bushing under simulated degradation: (**a**) impedance magnitude and (**b**) parallel capacitance curves.

The resulting frequency response curves for impedance magnitude Z(f) and parallel capacitance $C_{p}\left(f\right)$ are presented in Figure 11. The impedance magnitude curves Z(f) remain almost unchanged across the analyzed frequency range, indicating that the bushing’s inductive and resistive components are not significantly affected by the simulated degradation scenarios.

Conversely, the parallel capacitance curves $C_{p}\left(f\right)$ exhibit clear and progressive deviations in the mid-frequency range (10 kHz–1 MHz) as the additional capacitive elements (4.7 nF, 8 nF, and 10 nF) are introduced. These deviations are characterized by an upward shift in the capacitance baseline, reflecting the effect of the parallel capacitive paths created during the fault simulation.

In the bushing tests, adding 4.7 nF to the $C_{2}$ path resulted in a decrease in the measured parallel capacitance by approximately 4.1% in the 10–200 kHz range, although the impedance magnitude exhibited only subtle changes. Similarly, adding 10 nF to the $C_{2}$ path increased the measured parallel capacitance by about 7% at 50 kHz and nearly 10% in the 100–200 kHz range, again with only minor variations in impedance magnitude. These changes in $C_{p}\left(f\right)$ are consistent with early-stage dielectric degradation mechanisms, such as moisture ingress or partial discharge activity within the bushing insulation layers.

Furthermore, these capacitance variations become more evident at higher frequencies, where localized dielectric defects and surface contamination exert greater influence on the bushing’s electrical behavior. This frequency-dependent sensitivity makes $C_{p}\left(f\right)$ a more reliable indicator than Z(f) for the early detection of insulation anomalies in capacitive bushings.

It is important to note that, in this work, bushing degradation was modeled as a reduction in the equivalent capacitance scenario compatible with loss of active dielectric area, delaminations, or cracks. Under such incipient conditions, only small negative variations are expected, typically of a few percent. This experimental design was intentional, aiming to assess the sensitivity of FRACTIVE^®^ 4.0 in detecting subtle capacitance decreases, consistent with what has been observed in the field during the early stages of degradation [27].

The small changes observed in Figure 11 are consistent with the fact that the simulated degradation (parallel capacitors connected to emulate leakage paths) represented an incipient fault scenario, which naturally produces subtle deviations in the frequency response. This was intentional, since FRACTIVE^®^ 4.0 is designed to detect early-stage anomalies that may not be evident through conventional offline tests. In practical applications, long-term monitoring enhances sensitivity, as progressive deterioration amplifies these deviations over time. Therefore, the minor changes confirm both the realism of the simulation and the importance of continuous online monitoring to capture fault evolution.

### 3.3. Random Forest Results

**Figure 12:**
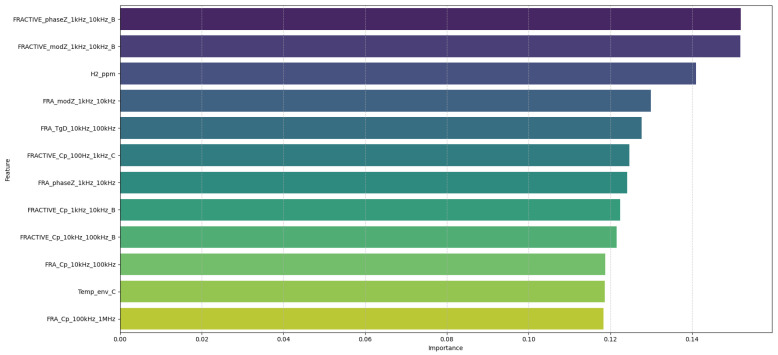
Top 12 original features ranked by contribution via PCA loadings, based on principal components retaining 20% of the total variance.

The contribution of the original features to the principal components used for training was assessed to identify the variables most influential in capturing the dataset variance. Figure 12 presents the top 12 original features ranked by their contribution via PCA loadings (20% of the total variance). This provides insight into which measured variables most strongly distinguish normal from anomalous conditions and enhances the interpretability of the Random Forest model.

Using these principal components as input, the Random Forest classifier was trained and evaluated on a held-out test set. As shown in Table 3, the classifier achieved perfect performance, with precision, recall, and F1-score values of 1.00 for both the normal and anomalous classes, and an overall accuracy of 100%. This ideal performance is a consequence of the controlled, simulated nature of the dataset, where distinctions between normal and anomalous conditions are well-defined and free of noise.

**Figure 13:**
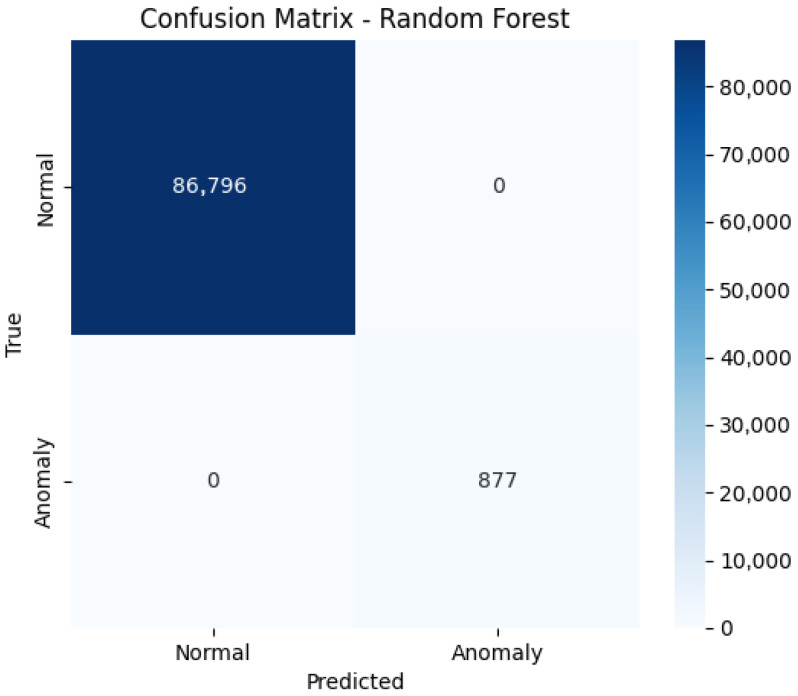
Confusion matrix for the Random Forest classifier, showing perfect separation between normal and anomalous samples.

The confusion matrix in Figure 13 further confirms the robustness of the model. All 86,796 normal samples were correctly classified as normal, and all 877 anomalous samples were correctly identified as anomalies. No false positives or false negatives were observed.

**Figure 14:**
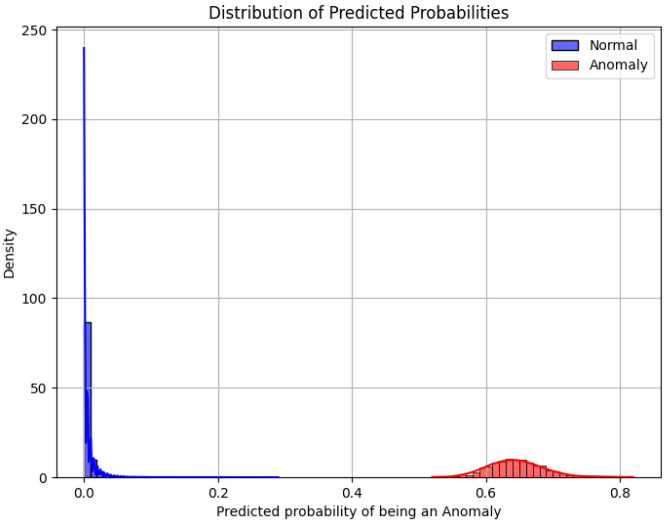
Distribution of anomaly scores predicted by the Random Forest model for normal and anomalous samples.

The probability distribution of predictions for both classes is shown in Figure 14. Normal samples are assigned probabilities of anomaly close to zero, while anomalous samples are assigned probabilities centered around 0.6. There is no overlap between the two distributions, which indicates that the model assigns well-separated and discriminative scores.

The clear separation between the probability distributions demonstrates that the classifier not only achieves correct binary predictions but also provides confidence scores that are well calibrated and reliable. This capability is relevant for applications that require uncertainty quantification, such as risk-based anomaly prioritization or adaptive decision thresholds.

Although such perfect results are rarely observed in real-world applications, it is expected that the model will still achieve high accuracy when applied to operational data. In practice, factors such as measurement noise, equipment aging, and operational variability may introduce slight deviations in performance; however, the strong separability observed in the simulated dataset suggests that the model can maintain robust predictive capability under realistic conditions.

## 4. Conclusions

Power transformers are critical assets in electrical power systems, and their failures can lead to widespread outages and significant economic losses. Continuous multi-sensor monitoring is therefore essential to detect incipient faults and prevent catastrophic events.

This work presented an integrated multi-sensor monitoring framework for power transformers, combining OnFRA^®^ 4.0 for frequency response analysis, FRACTIVE^®^ 4.0 for capacitive bushing monitoring, continuous gas and temperature measurements, and acquisition of electrical parameters. Laboratory experiments allowed for the controlled simulation of common fault scenarios, such as inter-turn short circuits, bushing insulation degradation, and variations in capacitance paths. These simulations provided a high-fidelity dataset that allowed the initial validation of anomaly detection algorithms in the absence of real operational failures.

Principal Component Analysis (PCA) was applied to reduce the dimensionality of the collected data, retaining components responsible for 20% of the total variance. This approach not only improved computational efficiency but also highlighted the most influential features contributing to anomaly detection, enhancing the interpretability of the machine learning model. The Random Forest classifier trained on these principal components achieved perfect performance on the simulated dataset, with all normal and anomalous samples correctly classified. The probability distribution of the predicted scores further confirmed the discriminative capacity of the model, demonstrating clear separation between classes.

The field implementation of the monitoring system confirmed its practical feasibility. Continuous data acquisition, integration with a SCADA supervisory platform, and real-time visualization of electrical, thermal, and diagnostic parameters were successfully achieved. The system demonstrated robustness to operational constraints, such as electromagnetic interference and environmental variability, providing a scalable framework for continuous monitoring and potential future expansion with additional sensors or analytical modules.

The main contribution of this work lies in the integration of online FRA with conventional diagnostic techniques for power transformers within a machine learning framework. Although FRA has been widely studied in offline applications, its use in online transformer monitoring is virtually unexplored in the practical literature, making this study one of the first to demonstrate its feasibility in a real-world context. Therefore, the proposed solution not only validates the potential of online FRA as a key variable for fault detection but also highlights its value in advancing predictive maintenance strategies in the context of digital substations.

Overall, this study demonstrates that the combination of high-fidelity sensor measurements with advanced machine learning techniques enables reliable detection of transformer anomalies under controlled conditions. While real in-service fault data are not yet available, the proposed methodology establishes a strong foundation for predictive maintenance in Substation 4.0 environments. The results highlight the potential of this integrated approach to enhance transformer reliability, reduce unplanned outages, and support data-driven maintenance decision-making.

## Figures and Tables

**Table 1:** General and specific characteristics of the transformer.

Characteristic	Value
Nominal power	ONAN: 121,800 kVA ONAF1: 162,400 kVA ONAF2: 200,000 kVA
Nominal voltage	High Voltage: 345,000 V Low Voltage: 15,000 V
Nominal current	High Voltage: 335 A Low Voltage: up to 7693.7 A
Nominal frequency	60 Hz
Connection configuration	Star/Delta (YNd11)
Type of insulating oil	Mineral—full vacuum resistant
Oil volume	42,860 L
Type of cooling	ONAN/ONAF (with voltage-free tap changer)
Short-circuit impedance	Total reactance: 15% No-load losses: 1500 kW
Excitation current	3.2% at 120% of nominal voltage
Short-circuit withstand capacity	Symmetrical: 40 kA Asymmetrical: 63 kA

**Table 2:** Common fault modes, affected frequency ranges, and typical FRA signatures (OnFRA^®^ 4.0).

Failure Mode	Frequency Range	Effect on Z(f)	Effect on ∠Z(f)
Inter-turn short circuit (ITSC)	10 kHz–1 MHz	Local resonance flattening, attenuation of impedance valleys	Mid-band phase deviation; loss of smooth transitions
Axial winding displacement	1 kHz–100 kHz	Shift of resonance peaks toward lower frequencies	Progressive phase lag in low-mid range
Radial deformation/coil bulging	100 kHz–3 MHz	New or distorted peaks; high-frequency ripple	Phase oscillations or instability in high-frequency region
Loose winding clamping	500 kHz–5 MHz	Local spectral noise; minor random peak displacements	Irregular fluctuations in phase beyond 1 MHz
Winding insulation thermal aging	10 kHz–1 MHz	Smooth impedance damping across midband	Phase shift trend across multiple ranges
Open interconnection/broken lead	<10 kHz	Sharp impedance rise; baseline displacement	Abrupt early phase drop; resonance suppression
Core-ground path degradation	100 Hz–5 kHz	Low-frequency impedance elevation	Flattening or dip in very low-frequency phase

**Table 3:** Classification report for the Random Forest model.

Class	Precision	Recall	F1-Score	Support
Normal	1.00	1.00	1.00	86,796
Anomaly	1.00	1.00	1.00	877
Accuracy	-	-	1.00	87,673
Macro avg	1.00	1.00	1.00	87,673
Weighted avg	1.00	1.00	1.00	87,673

## Data Availability

The data presented in this study are available on request from the corresponding author.

## Abbreviations

The following abbreviations are used in this manuscript:

EPS	Electrical Power System
AI	Artificial Intelligence
ANN	Artificial Neural Network
SVM	Support Vector Machine
RF	Random Forest
PCA	Principal Component Analysis
DGA	Dissolved Gas Analysis
PLC	Programmable Logic Controller

## References

[1] Dapshima B.A., Essa Y.C., Chaturvedi S. (2023). Fault detection and protection of power transformer using fuzzy logic. Int. J. Res. Appl. Sci. Eng. Technol. (IJRASET).

[2] Key S., Son G.W., Nam S.R. (2024). Deep learning-based algorithm for internal fault detection of power transformers during inrush current at distribution substations. Energies.

[3] Badawi M., Ibrahim S.A., Mansour D.E.A., El-Faraskoury A.A., Ward S.A., Mahmoud K., Lehtonen M., Darwish M.M. (2022). Reliable estimation for health index of transformer oil based on novel combined predictive maintenance techniques. IEEE Access.

[4] Viotti I.D., Ribeiro R.F., Gomes G.F. (2024). Damage identification in sandwich structures using Convolutional Neural Networks. Mech. Syst. Signal Process..

[5] Zhang J., Wang Y., Yang Y., Ma Y., Dai Z. (2024). Fault diagnosis and intelligent maintenance of industry 4.0 power system based on internet of things technology and thermal energy optimization. Therm. Sci. Eng. Prog..

[6] Auronen T., Murat I., Hanninen T., Keitoue S. (2020). Future trends in transformer online monitoring. Proceedings of the 5th International Colloquium on Transformer Research and Asset Management.

[7] Senobari R.K., Sadeh J., Borsi H. (2018). Frequency response analysis (FRA) of transformers as a tool for fault detection and location: A review. Electr. Power Syst. Res..

[8] Safari A., Sabahi M., Oshnoei A. (2024). ResFaultyMan: An intelligent fault detection predictive model in power electronics systems using unsupervised learning isolation forest. Heliyon.

[9] Žarković M., Stojković Z. (2017). Analysis of artificial intelligence expert systems for power transformer condition monitoring and diagnostics. Electr. Power Syst. Res..

[10] Vasconcelos G.A.V.B., Francisco M.B., da Costa L.R.A., Ribeiro Junior R.F., Melo M.d.L.N.M. (2024). Prediction of surface roughness in duplex stainless steel face milling using artificial neural network. Int. J. Adv. Manuf. Technol..

[11] Gomes G.F., Junior R.F.R., Pereira J.L.J., Francisco M.B. (2023). An efficient deep learning model to predict the structural response of CFRP isogrid tubes. Compos. Struct..

[12] Kakolaki S.E.H., Hakimian V., Sadeh J., Rakhshani E. (2023). Comprehensive study on transformer fault detection via frequency response analysis. IEEE Access.

[13] Zacarias T.G., Martins R., Xavier C.E., Castioni J.C.O., Sant’Ana W.C., Lambert-Torres G., Gama B.R., Areias I.A.D.S., Bonaldi E.L., Assuncao F.D.O. (2023). Detection of failures in metal oxide surge arresters using frequency response analysis. Sensors.

[14] Sant’Ana W.C., Lambert-Torres G., Bonaldi E.L., Gama B.R., Zacarias T.G., Areias I.A.D.S., Arantes D.D.A., Assuncao F.D.O., Campos M.M., Steiner F.M. (2021). Online frequency response analysis of electric machinery through an active coupling system based on power electronics. Sensors.

[15] Pramanik S., Satish L. (2015). A critical review of the definition of FRA resonance frequency of transformers as per IEEE Std C57. 149-2012. Electr. Power Syst. Res..

[16] Hashemnia N., Abu-Siada A., Islam S. (2015). Improved power transformer winding fault detection using FRA diagnostics—Part 1: Axial displacement simulation. IEEE Trans. Dielectr. Electr. Insul..

[17] Mitiche I., McGrail T., Boreham P., Nesbitt A., Morison G. (2021). Data-driven anomaly detection in high-voltage transformer bushings with LSTM auto-encoder. Sensors.

[18] Nuruzzaman M., Limon G.Q., Chowdhury A.R., Khan M.M. (2025). Predictive Maintenance in Power Transformers: A Systematic Review Of AI And IOT Applications. ASRC Procedia Glob. Perspect. Sci. Scholarsh..

[19] Khan M.M. (2025). AI and machine learning in transformer fault diagnosis: A systematic review. Am. J. Adv. Technol. Eng. Solut..

[20] Ramesh J., Shahriar S., Al-Ali A., Osman A., Shaaban M.F. (2022). Machine learning approach for smart distribution transformers load monitoring and management system. Energies.

[21] Breiman L. (2001). Random forests. Mach. Learn..

[22] Pearson K. (1901). On lines and planes of closest fit to systems of points in space. Philos. Mag..

[23] Hotelling H. (1933). Analysis of a complex of statistical variables into principal components. J. Educ. Psychol..

[24] Wold S., Esbensen K., Geladi P. (2018). Principal component analysis. Chemom. Intell. Lab. Syst..

[25] Zhu N., Zhu C., Zhou L., Zhu Y., Zhang X. (2022). Optimization of the random forest hyperparameters for power industrial control systems intrusion detection using an improved grid search algorithm. Appl. Sci..

[26] Teodorescu V., Obreja Brașoveanu L. (2025). Assessing the Validity of k-Fold Cross-Validation for Model Selection: Evidence from Bankruptcy Prediction Using Random Forest and XGBoost. Computation.

[27] (2012). IEEE Guide for Field Testing and Evaluation of the Insulation of Shielded Power Cable Systems Rated 5 kV and Above.

